# Physiological biodistribution on Ga68-PSMA PET/CT and the factors effecting biodistribution

**DOI:** 10.1007/s12149-024-01957-x

**Published:** 2024-07-09

**Authors:** Ayça Arçay Öztürk, Metin Erkılıç, Gonca Gül Bural, Funda Aydın, Adil Boz

**Affiliations:** Nuclear Medicine Department, Faculty of Medicine, Akdeniz University, Antalya, Turkey

**Keywords:** Prostate cancer, PSMA PET, Prostate specific membrane antigen, PSMA-617

## Abstract

**Aim:**

The study aims to determine the physiological and pathophysiological distribution of the radiopharmaceutical (Ga^68^-PSMA-617) and investigate whether there are differences in distribution according to the laboratory, histopathological and clinical findings that can affect image evaluation. Also, we aimed to determine cut-off values to distinguish physiological and pathological uptake in prostate, bone, and lymph nodes.

**Materials and Methods:**

229 prostate cancer patients who underwent Ga^68^-PSMA PET/CT at our department were retrospectively analyzed. The patients were grouped according to PET/CT results, Gleason scores, PSA values, received treatments, metastatic status and other laboratory values. The SUV values of the organs, tissues, and pathological lesions of the patients in these subgroups were compared among themselves.

**Results:**

No significant difference was detected in the physiological uptake of lymph nodes and bone between the groups. In the group with patients that received androgen deprivation therapy (ADT), the bone metastasis SUV values were found to be higher and the SUV values of the submandibular gland and renal cortex were found to be lower (Mann–Whitney *U*, *p* = 0.043; 0.004; 0.01, respectively). In the group with patients who received radiotherapy, the normal prostate tissue SUV values were determined to be higher (Mann–Whitney *U*, *p* = 0.009). The SUV values of the submandibular gland, muscle, liver, and blood pool were found to be lower in the group of patients with high serum LDH values. The cut-off SUVmax value was determined to be 6.945 (sensitivity 89.6%, specificity 98.1%) for primary prostate lesion; 4.72 for lymph node metastasis; 4.25 for bone metastasis. The serum PSA cut-off value to distinguish the negative/positive groups was found to be 1,505 (sensitivity 79.7%, specificity 77.3%).

**Conclusion:**

In conclusion, PSMA-617 demonstrates a similar biodistribution with other PSMA ligands. The physiological uptake of lymph nodes and bone which are mostly metastasized in prostate cancer, are not affected by the factors we examined. It should be kept in mind that the normal prostate tissue uptake may increase in patients receiving radiotherapy, and the physiological/pathological uptake of the organs may differ due to the changes in PSMA expression in patients receiving ADT, tumor burden, and kidney function may affect the biodistribution.

**Supplementary Information:**

The online version contains supplementary material available at 10.1007/s12149-024-01957-x.

## Introduction

Prostate cancer is the second most frequent cancer and the fifth leading cause of cancer death among men in 2020 [1]. Recently Ga^68^-PSMA PET/CT has been introduced, targeting prostate-specific membrane antigen (PSMA) which is a cell surface transmembrane protein overexpressed in prostate cancer cells [2]. It is increasingly widely used in primary staging, biochemical recurrence, and therapy response evaluation of prostate cancer. There are studies regarding the biodistribution of Ga^68^-PSMA-11 and Ga^68^-PSMA-I&T in the literature [3], [4], [5]. Preclinical studies showed that the binding affinity and internalization fraction of PSMA-617 ligand into prostate cancer cells are significantly high. In addition, it was observed that the tumor/background activity ratio after 24 h of the injection increased up to 1,508 [6]. In the clinical study conducted with PSMA-617 in 19 prostate cancer patients, the distribution and uptake of the tracer compared between the images obtained at different periods after injection and the radiation exposure by the radioligand was evaluated [7]. To the best of our knowledge, this is the first study to investigate the physiological and pathophysiological distribution of Ga^68^-PSMA-617 in a large patient group.

It is imperative to possess a comprehensive understanding of the physiological and pathophysiological distribution patterns of radiopharmaceuticals within normal organs and tumor lesions. This knowledge, coupled with an awareness of the influencing factors, is pivotal for refining the precision of PET scan evaluations. Furthermore, considering that factors affecting biodistribution in PSMA PET/CT may parallel those encountered during PSMA radionuclide therapy, being cognizant of these variables and aligning treatment strategies accordingly holds promise for optimizing therapeutic outcomes. Therefore, our study aims to determine the physiological and pathophysiological distribution of the radiopharmaceutical Ga^68^-PSMA-617 by detecting the range of uptake in the organs and tissues, primary prostate tumor, lymph node, and bone metastasis and investigate whether there are differences in distribution according to the laboratory, histopathological and clinical findings that can affect image evaluation of Ga^68^-PSMA-617 PET/CT in prostate cancer patients. Furthermore, we aimed to establish cut-off uptake values to distinguish primary prostate tumor from normal prostate tissue, bone and lymph node metastasis from physiological bone and lymph node uptake.

## Materials and methods

We retrospectively evaluated 229 prostate cancer patients who underwent Ga^68^-PSMA PET/CT in our department. Medical data in terms of patients’ age, received treatments, histopathological (Gleason score) and laboratory findings were extracted from the institutional database. Serum PSA, LDH, ALP, and creatinine levels within the same month of PET/CT were recorded.

This retrospective study was approved by the institutional ethics committee and written informed consent was obtained from all patients.

### PET/CT protocol

Ga^68^-chloride was obtained by elution of a Ge^68^/Ga^68^ generator (iThemba Labs, 1110 MBq reference activity) and 25 µg of PSMA-617 was labeled with Ga^68^-chloride solution by a fully automated radiopharmaceutical synthesis device (Scintomics). The radiochemical purity of Ga^68^-labeled PSMA conjugates was over 95%, based on radio-high performance liquid chromatography. Patients were injected intravenously with an average of 141 ± 25.5 (92.5–210.9) MBq Ga^68^-PSMA. An iodine-based contrast agent was administered orally to all patients. Ga^68^-PSMA PET/CT was performed on a dedicated PET/CT scanner (Biograph 16, Siemens, Erlangen, Germany) at 45–60 min after the injection. A low-dose CT scan (slice thickness 5 mm, 120 mAs, 130 kV) was obtained and used for anatomical correlation and attenuation correction. After the transmission scan, PET images were acquired for 3 min per bed position for 6–8 beds from the vertex to the upper thigh. PET images were reconstructed by the iterative method using ordered-subset expectation maximization. The reconstructed PET images, CT images, and fused PET/CT images were reviewed using the dedicated workstation (Syngo.via, Siemens).

### Evaluation of images

The images were reviewed by two experienced nuclear medicine physicians. Ga^68^-PSMA uptake in each organ above physiological background activity was considered pathological by visual evaluation.

To evaluate the physiological uptake of organs and tissues, regions of interest (ROI) were drawn from attenuation-corrected transaxial PET images by correlating them with CT images and semiquantitative parameters including maximum standard uptake value (SUVmax) and mean standard uptake value (SUVmean) within these areas were obtained. SUV values were obtained from 26 normal anatomical structures for each patient, avoiding the inclusion of any activity from adjacent organs. ROIs were drawn from the cerebral cortex (parietal lobe gray matter), cerebellar cortex, cranium, lacrimal gland, palatine tonsil, parotid gland, submandibular gland, larynx (vocal cord), nasopharynx, thyroid gland, lung (upper lobes peripheral regions), mediastinal lymph node, periareolar breast tissue, pancreas (corpus), spleen, renal cortex, jejunum, adrenal gland, stomach (corpus wall), bone marrow (iliac bone medullary region), bladder lumen, seminal vesicle, rectum wall, gluteus maximus muscle, subcutaneous adipose tissue (thigh region) and testis. ROIs were drawn from the regions of prostate tissue that don’t have pathological activity above the physiological background activity and the values obtained from these ROIs were considered normal prostate tissue values. Apart from these, in patients with degenerative bone changes, ROIs were drawn from the degenerative bone lesions (osteophytes) with the highest SUV values. ROIs were drawn from the lymph node and bone metastases with the highest SUV values if there was more than one bone or lymph node metastasis in the same patient.

Additionally, volumetric areas of interest (VOI) which were determined automatically by the Syngo.via workstation were drawn in the form of a spherical (2.75 cm in diameter) for the right lobe of the liver and cylindrical (0.75 cm in diameter and 2 cm in height) for the thoracic descending aorta, SUVmean values were obtained from these VOIs (Supplemental Fig. 1). ROIs from all regions (except the automated VOIs) were determined as areas smaller than 2 cm^2^.

**Fig. 1:**
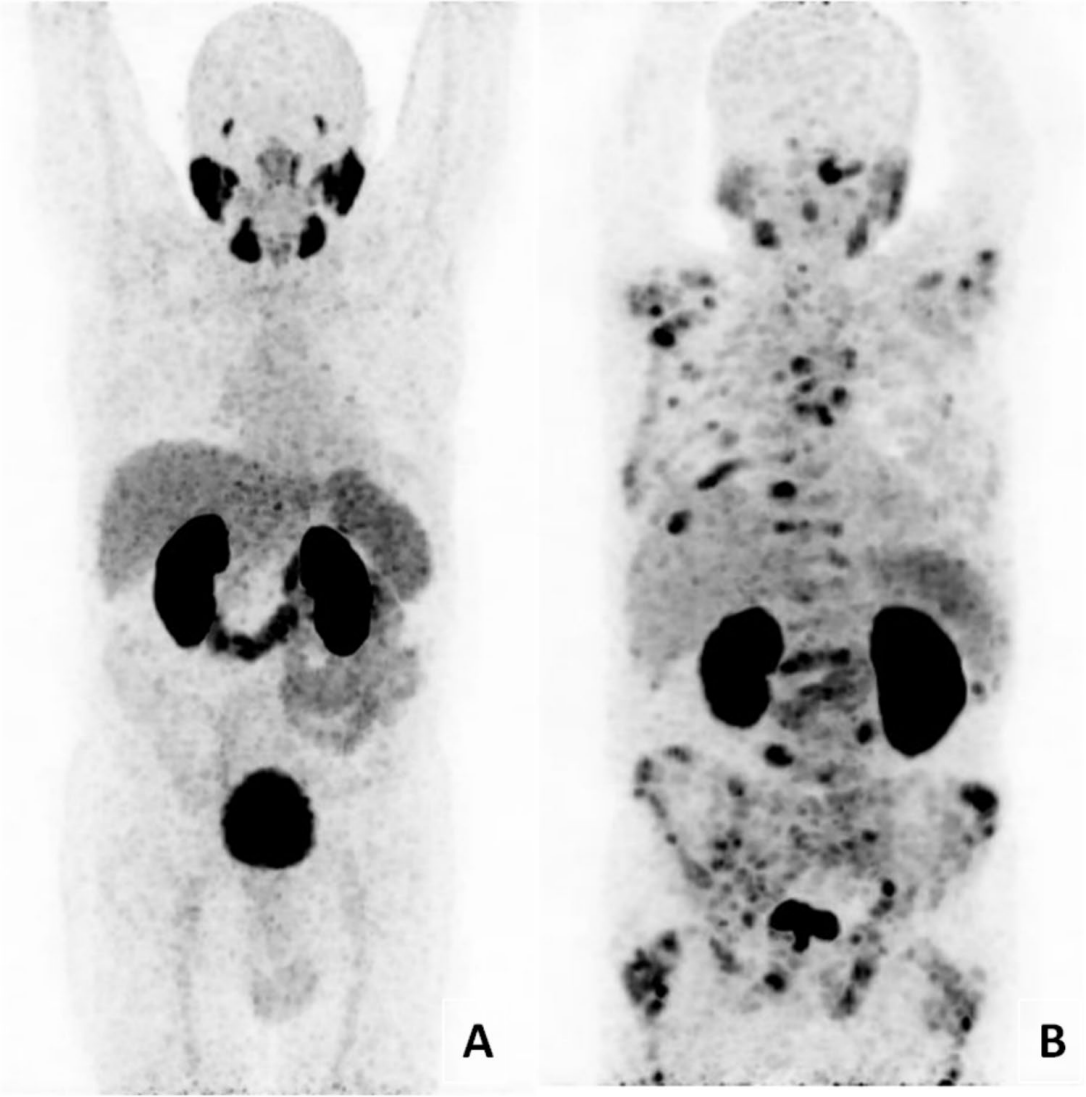
Example MIP images of patients belonging to negative (**A**) and positive (**B**) groups

Patients with no pathological activity on Ga^68^-PSMA PET/CT were considered as ‘negative group’, and the patients with pathological activity (primary prostate tumor, lymph node, or bone metastasis) on Ga^68^-PSMA PET/CT were considered as ‘positive group’ (Fig. 1). Within the positive group, subgroups were formed according to Gleason scores (differentiation degree grouping), PSA values (< 2 ng/ml, 2–20 ng/ml and > 20 ng/ml), therapy given before imaging (androgen deprivation therapy/ radiotherapy given/not given), metastatic status (with/without bone metastasis, with/without lymph node metastasis), ALP values (elevated/normal), LDH values (elevated/normal), serum creatinine values (elevated/normal). SUV values from these subgroups were compared among themselves within the positive group and then also with the negative group.

Utilizing SUVmean instead of SUVmax provides a more accurate assessment of overall activity, as it accounts for the average uptake within the entire region of interest rather than focusing on a limited, potentially non-representative segment. Therefore, in comparisons with subgroups, it was considered significant only when SUVmax and SUVmean values for the same organ revealed statistically significant differences at the same time to reduce the effect of heterogeneity and provide a more comprehensive assessment of activity. Isolated organ metastasis apart from the lymph node and bone was not observed in any patient.

### Statistical analysis

Statistical analysis of the data was performed using the IBM SPSS Statistics 23.0 software (IBM Corp., Arnork, NY). Number and percentage values were used to describe categorical data; while mean, standard deviation, median, minimum and maximum values were used to describe continuous data. The normality of the distribution of continuous variables was determined using the Kolmogorov–Smirnov test. In the comparison of two independent groups of continuous variables, the independent samples *t* test was used for normally distributed variables, and the Mann–Whitney *U* test was used for non-normally distributed variables. The differences among three and more independent groups were compared using one-way ANOVA with the Tukey post hoc test for normally distributed variables and the Kruskal–Wallis test with Bonferroni correction for non-normally distributed variables. Pearson Chi-Square test was used for the analysis of categorical variables. A *p* value of < 0.05 was considered statistically significant. Receiver operating characteristic (ROC) curve analyses including calculation of the area under the curve (AUC) were generated and the optimal cut-off values for each parameter were evaluated based on the Youden index.

## Results

A total of 229 patients’ and negative, positive group characteristics are summarized in Table 1.

**Table 1:** Characteristics of patients

	Total patients (*n* = 229)	Negative group (*n* = 66)	Positive group (*n* = 163)
Age mean ± SD	68.73 ± 7.96	68.5 ± 7.78	68.82 ± 8.05
Indication of PET/CT	Primary staging: %35.3 (*n* = 81) Restaging: %42.3 (*n* = 97) Therapy response evaluation: %22.2 (*n* = 51)	Primary staging: %40.9 (*n* = 27) Restaging: %40.9 (*n* = 27) Therapy response evaluation: %18.2 (*n* = 12)	Primary staging: %33.1 (*n* = 54) Restaging: %42.9 (*n* = 70) Therapy response evaluation: %23.9 (*n* = 39)
Gleason Grade Group (*n* = 131, *n* = 43, *n* = 88, respectively)	Group 1: %19.8 (*n* = 26) Group 2: %22.9 (*n* = 30) Group 3: %12.2 (*n* = 16) Group 4: %17.5 (*n* = 23) Group 5: %27.4 (*n* = 36)	Group 1: %23.3 (*n* = 10) Group 2: %30.2 (*n* = 13) Group 3: %11.6 (*n* = 5) Group 4: %16.2 (*n* = 7) Group 5: %18.6 (*n* = 8)	Group 1: %18.1 (*n* = 16) Group 2: %19.3 (*n* = 17) Group 3: %11.4 (*n* = 11) Group 4: %18.2 (*n* = 16) Group 5: %31.8 (*n* = 28)
PSA in ng/ml (*n* = 91, *n* = 22, *n* = 69, respectively)	Mean ± SD = 39.53 ± 107.2 Range: 0.01–699.79	Mean ± SD = 3.77 ± 8.73 Range: 0.01–38	Mean ± SD = 50.94 ± 121 Range: 0.05–669.79
LDH in U/L (*n* = 68, *n* = 13, *n* = 55, respectively)	Mean ± SD = 264.5 ± 134.84 Range: 138–945	Mean ± SD = 227.69 ± 51.22 Range: 144–326	Mean ± SD = 273.26 ± 146.82 Range: 138–945
ALP in IU/L (*n* = 59, *n* = 9, *n* = 50, respectively)	Mean ± SD = 159.97 ± 192.48 Range: 36–995	Mean ± SD = 84.22 ± 14.74 Range: 65–118	Mean ± SD = 173.6 ± 206.34 Range: 36–995
Serum creatinine in mg/dl (*n* = 91, *n* = 21, *n* = 70, respectively)	Mean ± SD = 0.94 ± 0.3 Range: 0.37–2.12	Mean ± SD = 0.96 ± 0.24 Range: 0.56–1.62	Mean ± SD = 0.94 ± 0.31 Range: 0.37–2.12

### Biodistribution findings

Considering the physiological biodistribution in 229 patients, the highest Ga^68^-PSMA activity was found in descending order of the renal cortex (mean ± SD SUVmax 46.59 ± 14.77), bladder lumen (mean ± SD SUVmax 29.38 ± 25.09), submandibular gland (mean ± SD SUVmax 17.33 ± 4.79), parotid gland (mean ± SD SUVmax 17.14 ± 4.91), lacrimal gland (mean ± SD SUVmax 12.94 ± 4.86) and jejunum (mean ± SD SUVmax 12.56 ± 4.95). Moderate uptake of Ga^68^-PSMA was observed in the spleen (mean ± SD SUVmax 9.37 ± 3.23), liver (mean ± SD SUVmax 7.96 ± 2.58), and tonsil (mean ± SD SUVmax 6.09 ± 2.72); the other organs had lower activities of Ga^68^-PSMA. Mean ± SD, median, and range SUV values of every anatomical structure are given in Supplemental Table 1 and shown in Supplemental Fig. 2.

### Effect of factors on biodistribution

Comparisons of organ SUV values between subgroups (those receiving and not receiving ADT) within the positive group, as well as between the subgroup receiving ADT and the negative group were performed. The noteworthy common findings from these two comparisons are outlined below: the subgroup that received ADT exhibited significantly lower SUVmax and SUVmean values in the submandibular gland, renal cortex, and testis, along with lower SUVmean (automatized) values in the liver (Table 2).

**Table 2:** SUV and p values found to be significantly different between the subgroups that received and not received ADT in the positive group and the negative group

	Positive group ADT given subgroup (*n* = 77) Mean ± SD	Positive group ADT not given subgroup (*n* = 69) Mean ± SD	Negative group (*n* = 66) Mean ± SD	*p* values (between the first two groups and between the 1st and 3rd groups respectively)
Submandibular gland SUVmax	16.10 ± 4.65	17.68 ± 5.16	18.25 ± 4.22	*p* = 0.037 *p* = 0.003
Submandibular gland SUVmean	11.25 ± 3.52	12.92 ± 3.99	12.75 ± 3.39	*p* = 0.004 *p* = 0.004
Renal cortex SUVmax	42.22 ± 13.81	48.79 ± 15.82	50.68 ± 12.47	*p* = 0.01 *p* < 0.001
Renal cortex SUVmean	29.72 ± 10.01	34.31 ± 11.06	33.94 ± 8.43	*p* = 0.011 *p* = 0.007
Liver SUVmean (automatized)	4.10 ± 1.24	4.73 ± 1.36	4.64 ± 1.33	*p* = 0.019 *p* = 0.021
Testis SUVmax	2.21 ± 0.82	2.84 ± 0.92	2.94 ± 0.84	*p* < 0.001 *p* < 0.001
Testis SUVmean	1.40 ± 0.47	1.81 ± 0.56	1.91 ± 0.49	*p* < 0.001 *p* < 0.001

As radiotherapy primarily targets localized areas, a comparison between the subgroups that received and not received RT was performed only for the iliac bone, rectum, prostate tissue, and seminal vesicles SUV values. Notably, in the subgroup that underwent RT, normal prostate tissue exhibited higher SUVmax and SUVmean values (SUVmax mean ± SD 4.68 ± 1.31; 3.9 ± 1.32; *p* = 0.009, SUVmean mean ± SD 3.08 ± 0.79; 2.71 ± 0.89; *p* = 0.037).

Additionally, we observed significant differences in blood pool SUVmean (automatized) and muscle SUVmax and SUVmean values between subgroups categorized by serum LDH levels. The subgroup with high (≥ 246 U/L) serum LDH values displayed lower values in blood pool SUVmean (1.87 ± 0.62; 2.31 ± 0.54; *p* = 0.006), muscle SUVmax (0.91 ± 0.44; 1.12 ± 0.39; *p* = 0.025), and muscle SUVmean (0.54 ± 0.21; 0.65 ± 0.2; *p* = 0.031) compared to the subgroup with normal (< 246 U/L) LDH values.

Three subgroups for GS were formed in the positive group according to the degree of differentiation: well-differentiated (GS 6, *n* = 16), moderately differentiated (GS 7, *n* = 28), and poorly differentiated (GS 8,9 and 10, *n* = 44). No significant differences in organ SUV values were observed when comparing these three GS subgroups.

In the assessment of organ SUV values between subgroups with high (≥ 1.3 ng/ml) and normal (< 1.3 ng/ml) serum creatinine levels, the subgroup with elevated serum creatinine exhibited significantly higher values in submandibular gland SUVmax and SUVmean, blood pool, and liver SUVmean (automatized) (Table 3).

**Table 3:** SUV and p values found to be significantly different between the subgroups with high and normal serum creatinine levels

	Positive group High serum creatinine level subgroup *n* = 7 Mean ± SD	Positive group Normal serum creatinine level subgroup *n* = 63 Mean ± SD	*p* values
Submandibular gland SUVmax	21.99 ± 6.76	15.43 ± 4.15	*p* = 0.008
Submandibular gland SUVmean	15.94 ± 5.44	10.72 ± 2.99	*p* = 0.009
Liver SUVmean (automatized)	5.23 ± 0.95	4.08 ± 1.15	*p* = 0.011
Blood pool SUVmean (automatized)	2.49 ± 0.53	2.01 ± 0.56	*p* = 0.038

### Analysis of the pathological lesions and cut-off values

**Fig. 2:**
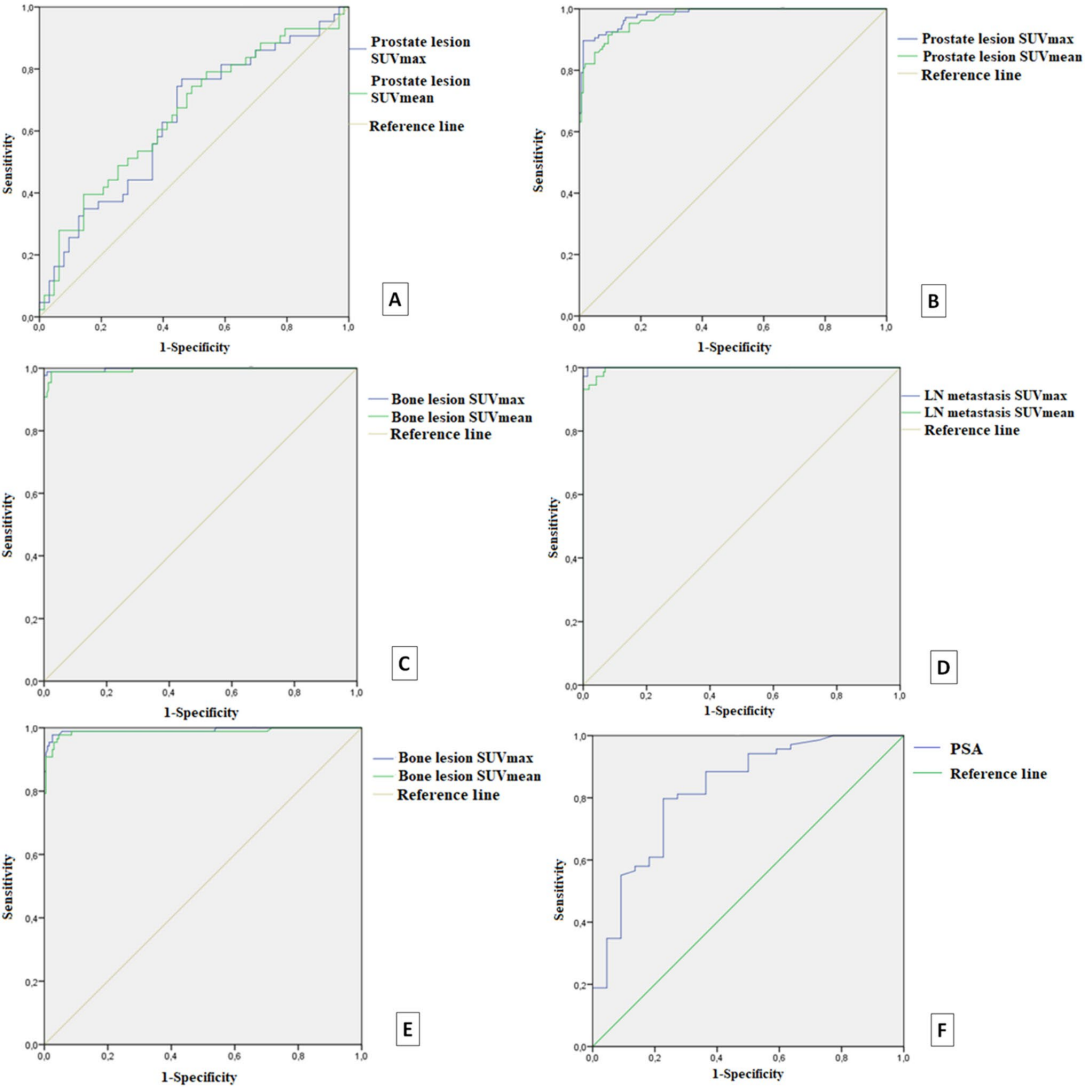
ROC analysis of SUV values of prostate lesions in the patients with and without lymph node metastasis (**A**), SUV values of prostate lesions in all patients (**B**), SUV values of bone lesions in all patients when the iliac bone was considered as negative reference (**C**), SUV values of lymph node metastasis in all patients (**D**), SUV values of bone lesions in all patients when degenerative bone lesions was considered as negative reference (**E**), PSA values in all pateints (**F**)

In comparison of the pathological lesion SUV values between the subgroups with and without lymph node metastasis, pathological prostate lesion SUVmax and SUVmean values were found to be significantly higher in the subgroup with lymph node metastasis (prostate lesion SUVmax 23.82 ± 19.03; 16.82 ± 13.75, *p* = 0.017, SUVmean 13.92 ± 11.52; 9.32 ± 7.77; *p* = *p* = 0.008). Receiver-operating-characteristic analysis revealed an optimal prostate lesion SUVmax cut-off of 12.385 to distinguish patients with and without lymph node metastasis (area under the curve 0.636, *p* < 0.001, 95% CI: 0.528–0.745), which gave 76.7% sensitivity and 54% specificity. ROC curve is given in Fig. 2.

Pathological bone lesion SUV values in the subgroup that received ADT demonstrated a significantly higher mean ± SD (*n* = 36, SUVmax 27.96 ± 19.59; SUVmean 14.94 ± 11.84) than the bone lesion SUV values in the subgroup that not received ADT (*n* = 56, SUVmax 18.36 ± 8.98; *p* = 0.043; SUVmean 8.7 ± 4.06, *p* = 0.002).

In comparison of the pathological lesion SUV values between the subgroups with high and normal serum ALP levels, pathological bone lesion SUVmax and SUVmean values were found to be significantly higher in the subgroup with high serum ALP levels (SUVmax 32.19 ± 24.51; 17.47 ± 10.91; *p* = 0.022, SUVmean 18.56 ± 16.63; 8.77 ± 5,85; *p* = 0.003).

Comparing the pathological lesion SUV values between the Gleason score subgroups demonstrated that the well-differentiated subgroup had significantly lower pathological prostate lesion SUV mean ± SD than the poorly differentiated subgroup (SUVmax 12.3 ± 8.7; 21.22 ± 13.97; *p* = 0.021, SUVmean 6.71 ± 3.92; 13.39 ± 11.58; *p* = 0.015). There was no significant difference between other subgroups.

ROC analysis revealed an optimal prostate lesion SUVmax and SUVmean cut-off of 6.945 and 3.89 for discrimination of pathological prostate lesions (*n* = 106) from normal prostate tissue (*n* = 160), which gave 89.6% sensitivity, 98.1% specificity (AUC: 0.892, 95% CI: 0.970–0.994) and 91.5% sensitivity, 90.6% specificity (AUC: 0.973, %95 CI: 0.958–0.988), respectively (b, Fig. 2).

In the ROC analysis SUVmax of 4.25 and SUVmean of 2.32 were the optimal cut-off values for distinguishing pathological bone lesion (*n* = 87) from physiological iliac bone (*n* = 216) with 98.9% sensitivity, 99.1% specificity (AUC: 0.998, 95% CI: 0.993–1) and 98.9% sensitivity, 97.7% specificity (AUC: 0.995, 95% CI: 0.989–1), respectively (c, Fig. 2). When degenerative bone lesions/changes were considered as non-pathological bone tissue and 198 degenerative bone lesion SUV values were analysed, SUVmax of 4.95 and SUVmean of 2.75 were the optimal cut-off values with 97.7% sensitivity, 97.5% specificity (AUC: 0.992, 95% CI: 0.980–1) and 97.7% sensitivity, %95.5 specificity (AUC: 0.988, 95% CI: 0.972–1), respectively (e, Fig. 2).

In the ROC analysis, SUVmax of 4.72 and SUVmean of 3.11 were the optimal cut-off values for distinguishing pathological lymph node (*n* = 73) from physiological mediastinal lymph node (*n* = 217) with 97.3% sensitivity, 100% specificity (AUC: 1, 95% CI: 0.999–1) and 93.2% sensitivity, 100% specificity (AUC: 0.997, 95% CI: 0.993–1), respectively (d, Fig. 2).

In the comparison of SUVmax values of the pathological prostate, lymph node, and bone lesions; the pathological bone lesions demonstrated a significantly higher mean than the pathological prostate lesions (26.09 ± 19.9, 19.65 ± 16.38, *p* = 0.015). There is no significant difference between pathological lymph node and prostate lesion SUVmax values (24.18 ± 25.66, 19.65 ± 16.38, *p* = 0.15) and between the lymph node and bone lesions SUVmax values (24.18 ± 25.66, 26.09 ± 19.9 *p* = 0.59).

### Analysis according to PSA values and gleason scores

There was a significant difference between negative and positive groups in terms of serum PSA value (*p* < 0.001). No statistically significant difference was found between positive and negative groups in terms of Gleason score (*p* = 0.069). In the ROC analysis for the study cohort, the optimal cut-off value of serum PSA was determined as 1.505 ng/ml for distinguishing negative and positive groups, with an area under the curve of 0.825 (95% CI: 0.721–0.928). ROC curve is given in f, Fig. 2.

## Discussion

It is crucial to be aware of the physiological and pathophysiological distribution of the radiopharmaceuticals in the normal organs and tumoral lesions and the factors that may affect the distribution to increase the accuracy of the PET scan evaluation. Furthermore, since effects similar to the factors that influence biodistribution on PSMA PET/CT may occur during PSMA radionuclide therapy, being aware of these factors and guiding the treatment accordingly may contribute to the effectiveness of the therapy.

Recently, there have been studies investigating the distribution pattern of Ga^68^-PSMA-11, Ga^68^-PSMA-I&T and reporting the mean, range of SUV values of the normal organs and tissues [3–5]. Although there are some minor differences in the ranking, the organs with the highest uptake and SUVmax values of the organs are similar to our study. In another study conducted with Ga^68^-PSMA-617 in 19 prostate cancer patients, the distribution and uptake on scans taken at different times after injection were compared and a dosimetry study was performed [7]. In the scans taken after 1 and 3 h after injection, the organs with the highest uptake were observed in the kidneys, salivary glands, lacrimal gland, small intestine, spleen, and liver in decreasing order and these findings are similar to our study. To the best of our knowledge, our study is the first to examine the physiological and pathophysiological distribution of Ga^68^-PSMA-617 in a large patient group.

The most common metastatic involvements of prostate cancer occur in the lymph nodes and bones. Since the physiological uptake of mediastinal lymph nodes and iliac bone demonstrated no significant difference between the groups and subgroups we examined, being aware that various laboratory and clinical factors don’t affect the physiological uptake of lymph nodes and bones may be useful for recognizing their pathological involvements of them.

Based on in vitro studies, it is known that ADT increases PSMA expression [8, 9]. Nevertheless, there is no consensus among imaging professionals. Although most of the studies have revealed that ADT increases PSMA expression, there are different results depending on short or long-term ADT, castration-resistant or castration-naive patients [10]. In a prospective study conducted with Ga^68^-PSMA-11 PET/MR images of 9 prostate cancer patients without previous treatment, a heterogenous increase of PSMA uptake which was more evident in bone metastases (average of 77% increase in SUVmax value) was observed on 34 weeks after ADT [11]. In our study, in the comparison of the subgroups (ADT given/not given) within the positive group, SUV values of the pathological bone lesions were found to be significantly higher in the subgroup that received ADT, no significant difference was found for pathological lymph node and prostate lesions. Nevertheless, the submandibular gland and renal cortex SUV values were significantly lower in the subgroup that received ADT than the negative group and the subgroup that not received ADT. Unlike the aforementioned prospective study, the evaluation in our study was performed not on the same patients, but on the different patients who received and didn’t receive ADT and without considering the time difference between therapy and scan. These may be the reason for the different results and it can be investigated with studies involving larger patient groups whether there is an increase in PSMA expression of physiological uptake of organs.

Testis SUV values were found to be significantly lower in the subgroup that received ADT when comparing both with the negative group and the subgroup that didn’t receive ADT. Medical castration causes testicular atrophy and a decline in the testicular size and weight. Testicular histology examination of the patients who received ADT revealed fibrosclerotic and atrophic changes [12]. The difference in testis SUV values may be related to this phenomenon.

The difference in normal prostate tissue SUV values between the subgroups that received and didn’t receive RT within the positive group was thought to be related to the inflammatory effects of RT. However, the time between RT and scan wasn’t considered in this study, therefore the accuracy and the reason for this result can be examined in larger patient groups. However, this finding should be taken into account as the high SUV values of the prostate tissue may affect the assessment of local recurrence after RT.

Submandibular SUV values, liver, and blood pool SUVmean (automatized) values were significantly higher in the subgroup with high serum creatinine levels than in the subgroup with normal serum creatinine levels. This result might be due to the increase in the amount of tracer remaining in the body because of the diminished excretion caused by kidney dysfunction.

Serum LDH level correlates with the tumor burden and was thought to reflect tumor growth and invasive potential. Elevated LDH may be a prognostic tumor marker in many solid tumors, including prostate cancer [13]. Tumor burden may affect the tracer biodistribution in normal organs due to the tumor sink effect. The study performed with 135 patients who underwent Ga^68^-PSMA-11 PET/CT for staging reported that radiopharmaceutical uptake in normal organs and tissues decreased by 58–64% in patients with high tumor burden. The decrease was observed especially in the kidneys, salivary glands, and lacrimal glands [14]. In the comparison of SUV values between the subgroups with high and normal LDH levels; blood pool SUVmean (automated) and muscle SUVmax and SUVmean values were found to be significantly lower in the subgroup with high LDH levels in the present study. These results are attributed to the tumor sink effect in the patients with high tumor burden. Additionally, these results may suggest that in the patients with high tumor burden, Lu^177^-PSMA therapy may be administrated at tolerably higher activities before side effects in dose-limiting organs such as kidneys and salivary glands become apparent.

Uprimny et al. reported that primary tumor SUVmax values were significantly higher in the patients with lymph node metastases than in the patients without lymph node metastases in 90 prostate cancer patients who underwent PET/CT for staging [15]. Similarly in our study, SUVmax and SUVmean values of the prostate lesion were found to be significantly higher in the patients with lymph node metastases despite that our study includes a more heterogenous patient group than the mentioned study with only staging patients. On the other hand, the same results weren’t observed between the groups with and without bone metastases in the aforementioned study and in our study. These findings might be due to the distinctions between the hematogenous and lymphogenous metastasizing primary tumors. Furthermore, the primary prostate lesion SUVmax and SUVmean optimal cut-off values for discrimination of the cases with and without lymph node metastases were found to be 12,385 and 6,945, respectively in this study.

Çağlar et al., in their study with 95 newly diagnosed or recurrent prostate cancer patients, found a moderate positive correlation between the number of bone metastasic regions on Ga^68^-PSMA PET/CT and serum ALP levels (*r* = 0.697, *p* < 0.001) [16]. The present study did not examine the correlation between the serum ALP levels and the number of metastatic regions, however, it revealed that pathological bone lesion SUV values were significantly higher in the patients with high serum ALP levels than the patients with normal serum ALP levels.

In a retrospective analysis of 21 prostate cancer patients who underwent Ga^68^-PSMA PET/CT before RP and histopathological examination after RP based on a 6 segments model, Fendler et al. reported SUVmax values of histopathology-positive segments were higher than histopathology-negative segments, ROC analysis revealed an optimal SUVmax cut-off value of 6.5 (67% sensitivity, 92% specificity, AUC: 0.84) to distinguish histopathologically positive and negative segments [17]. In our cohort, although histopathological confirmation couldn’t be obtained, ROC analysis of 106 pathological prostate lesions and 160 normal prostate tissue revealed an optimal cut-off SUVmax value of 6.945 (89.6% sensitivity, 98.1% specificity, AUC: 0.982) and it is similar to the study with histopathological confirmation.

Examination of the pathological prostate, lymph node, and bone lesions SUV values of the prostate cancer patients revealed different consequences. Some studies showed that there is no significant difference between the lesion SUV values [18, 19], whereas some demonstrated that the pathological bone lesions had significantly higher SUV values than the other lesions [7]. We also found that SUV values of the pathological bone lesions were significantly higher than the pathological lymph node and prostate lesions.

Analyses investigating whether the PSMA uptake of the primary tumor differs according to the clinical parameters revealed a significant difference in uptake between different PSA levels and Gleason score groups [15, 20]. In our cohort, analysis of the Gleason score groups showed that primary tumor SUV values were significantly lower in the well-differentiated group than in the poorly differentiated group. In this manner, the tendency of higher PSMA uptake in higher-grade malignancies reported in the literature was demonstrated in the present study. However, no significant difference in pathological prostate lesion SUV values was found between the PSA-level groups.

Recently, many studies have examined the role of Ga^68^-PSMA PET/CT in patients with recurrent prostate cancer. In these retrospective analyses, they reported significantly higher PSA levels in patients with positive findings on Ga^68^-PSMA PET/CT than the patients without positive findings on scan [7, 18, 19, 21, 22]. On the other hand, similarly to our cohort, the study conducted in a heterogeneous group of 415 patients who underwent PET/CT for staging, restaging, and biochemical recurrence, PSA values were found to be significantly higher in the patients with positive scans than the patients with negative scans [23]. Since the increase in PSA levels indicates progressive disease, these consequences are expected and the present study had the same results. In the mentioned studies, ROC analysis revealed the optimal PSA cut-off values for discrimination of positive and negative scans as follows: 0.83 (AUC: 0.868) in the study with 70 recurrent prostate cancer patients [18], 0.67 (95.6% sensitivity, 83.3% specificity, AUC: 0.952) in the study with 109 recurrent prostate cancer patients [22] and 1.16 (77% sensitivity, 75% specificity, AUC: 0.805) in the study with 415 patients [23]. In our cohort with 22 negative group patients and 69 positive group patients, an optimal PSA cut-off value of 1.505 was obtained with 79.7% sensitivity and 77.3% specificity (AUC: 0.825). It is higher than the other studies in the literature, however, it is closer to the cut-off value in the study with a heterogenous group of 415 patients similar to our study.

Afshar-Oromieh et al., in a retrospective analysis performed on 1007 patients who underwent Ga^68^- PSMA-11 PET/CT for recurrent prostate cancer, found that there is no significant difference in terms of Gleason scores between the patients with and without positive findings on scan [24]. Despite the most of the studies have found similar results [19, 22, 25], there are also studies reporting a significant difference [21, 23]. We ended up with no significant difference in Gleason score between the patients with and without pathological activity as reported in most of the studies in the literature.

Our study has inherent limitations because of its retrospective nature. The lack of histopathological confirmation of the lesions, the inaccessibility of the clinical and laboratory data for every patient, and the heterogeneous patient population are some of them. Moreover, the number of patients in some of the subgroups is low due to groupings despite the study has a relatively high patient number. Additionally, the absence of partial volume correction represents a significant limitation that is essential for accurate assessment of activity, particularly for small organs.

## Conclusion

In conclusion, PSMA-617 shows similar biodistribution to other PSMA ligands using for imaging. Physiological uptake of lymph nodes and bones, which are mostly metastasized in prostate cancer, are not affected by the factors we examined. It should be considered that normal prostate tissue SUV values may increase in patients receiving RT, physiological and pathological uptake of the organs may differ due to the changes in PSMA expression in patients receiving ADT, the tumor burden and the kidney function may influence the biodistribution.

## Supplementary Information

Below is the link to the electronic supplementary material.

Supplementary file1 (DOCX 555 KB)

## Data Availability

All data generated and analyzed during this study are included in this published article.
