# Supplementary material for: Physiological biodistribution on Ga68-PSMA PET/CT and the factors effecting biodistribution
Source: Ann Nucl Med. 2024 Jul 9;38(11):894–903. doi: 10.1007/s12149-024-01957-x (PMC11489224; doi:10.1007/s12149-024-01957-x)
Supplement: Supplementary file 1 — Supplementary file1 (DOCX 555 KB) [file 12149_2024_1957_MOESM1_ESM.docx]

Supplemental Table 1: Mean±SD, median ,and range SUV values of the organ

|  | Mean±SD SUVmax | Median  SUVmax | Mean±SD SUVmean | Median  SUVmean |
| --- | --- | --- | --- | --- |
| Cerebral cortex | 0,32±0,18 | 0,28  (0,06-1,02) | 0,13±0,07 | 0,12  (0,01-0,49) |
| Cerebellar cortex | 0,46±0,29 | 0,38  (0,06-1,97) | 0,22±0,11 | 0,19  (0,01-0,72) |
| Cranium | 1,13±0,54 | 1,02  (0,06-3,29) | 0,63±0,27 | 0,58  (0,02-1,51) |
| Lacrimal gland | 12,94±4,86 | 12,87  (0,83-28,86) | 5,82±2,26 | 5,71  (0,5-13,4) |
| Tonsil | 6,09±2,72 | 5,85  (1,28-16,4) | 3,68±1,49 | 3,56  (0,7-9,43) |
| Parotid gland | 17,14±4,91 | 17,33  (4,6-31,44) | 12,91±3,88 | 12,96  (3,09-24,22) |
| Submandibular gland | 17,33±4,79 | 16,83  (3,48-32,98) | 12,32±3,74 | 11,86  (2,7-23,71) |
| Larynx | 3,78±1,86 | 3,45  (0,95-13,53) | 2,46±1,08 | 2,23  (0,77-7,05) |
| Nasopharynx | 3,09±1,53 | 2,57  (0,81-8,3) | 1,65±0,72 | 1,5  (0,56-5,67) |
| Thyroid | 2,6±0,81 | 2,55  (0,74-4,99) | 1,73±0,51 | 1,68  (0,43-3,99) |
| Lung | 0,9±0,42 | 0,82  (0,24-3,03) | 0,55±0,23 | 0,52  (0,15-1,56) |
| Mediastinal lymph node | 1,97±0,67 | 1,91  (0,75-4,24) | 1,41±0,47 | 1,39  (0,34-3,02) |
| Blood pool | 3,08±0,92 | 2,99  (1,36-6,57) | 2,14±0,6 | 2,13  (1-4,98) |
| Breast tissue | 0,98±0,43 | 0,93  (0,28-3,21) | 0,6±0,29 | 0,57  (0,08-2,41) |
| Pancreas | 4,19±1,72 | 3,95  (0,94-16,1) | 2,84±1,12 | 2,63  (0,87-12,49) |
| Liver | 7,96±2,58 | 7,74  (2,14-17,69) | 5,43±1,73 | 5,23  (1,68-11,07) |
| Spleen | 9,37±3,23 | 8,82  (3,2-19,39) | 6,95±2,56 | 6,51  (0,36-14,91) |
| Renal cortex | 46,59±14,77 | 46,62  (16,55-89,63) | 32,27±10,28 | 32,24  (11,81-73,91) |
| Jejunum | 12,56±4,95 | 11,77  (1,67-30,81) | 8,38±3,12 | 8,21  (1,3-19,96) |
| Adrenal gland | 2,65±1,06 | 2,57  (0,6-7,4) | 1,91±0,69 | 1,84  (0,4-4,22) |
| Stomach | 3,26±1,38 | 3,07  (0,99-10,04) | 2,18±0,83 | 2,14  (0,65-5,82) |
| Bladder lumen | 29,38±25,09 | 22,23  (0,64-158,96) | 22,97±18,67 | 18,03  (0,44-116,15) |
| Prostate | 4,09±1,37 | 3,89  (1,54-10,12) | 2,79±0,87 | 2,66  (0,84-6,22) |
| Seminal vesicle | 2,61±1,25 | 2,4  (0,53-8,98) | 1,76±0,85 | 1,62  (0,36-6,95) |
| Rectum | 2,29±0,96 | 2,05  (0,65-6,01) | 1,6±0,66 | 1,52  (0,17-4,2) |
| Muscle | 1,04±0,44 | 0,98  (0,3-3,11) | 0,6±0,22 | 0,57  (0,2-1,78) |
| Subcutaneous adipose tissue | 0,49±0,2 | 0,43  (0,18-1,55) | 0,3±0,11 | 0,29  (0,1-0,82) |
| Testis | 2,63±0,93 | 2,56  (0,63-5,7) | 1,7±0,56 | 1,68  (0,42-4,29) |
| İliac bone | 1,47±0,68 | 1,35  (0,48-4,89) | 0,96±0,47 | 0,9  (0,3-3,42) |


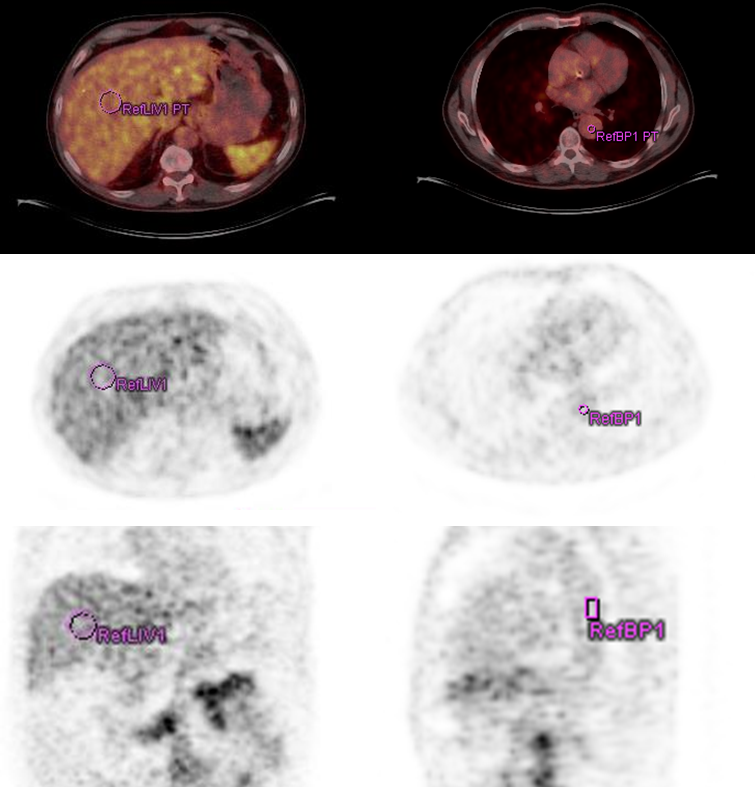


Supplemental Figure 1: VOIs of liver and blood pool automatically taken by Syngo.via workstation


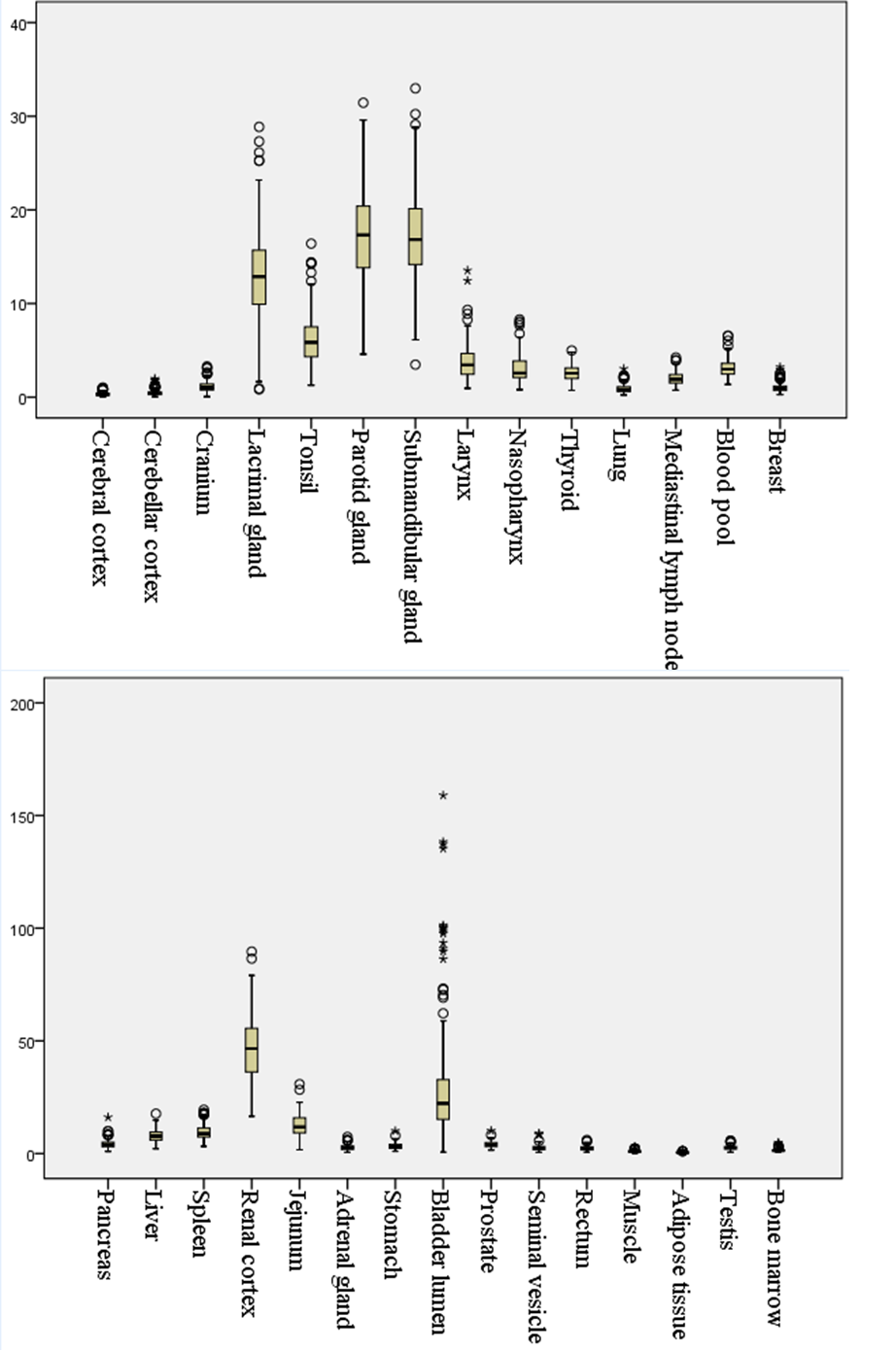


Supplemental Figure 2: Box plot of organ and tissues SUVmax values
